# Pass quantity, focus on quality

**DOI:** 10.1186/s13045-015-0123-7

**Published:** 2015-03-18

**Authors:** Delong Liu, Weijing Sun, Jingzhou Hou, Ruirong Yuan, Zihai Li

**Affiliations:** Division of Hematology and Oncology, New York Medical College, Valhalla, NY 10595 USA; Department of Medicine, Division of Hematology-Oncology, University of Pittsburgh Cancer Institute, Pittsburgh, PA 15232 USA; Division of Hematology-Oncology, VA Medical Center-UMDNJ, East Orange, NJ 07018 USA; Department of Microbiology and Immunology, Medical University of South Carolina, Charleston, SC 29425 USA

**Keywords:** Hematology, Oncology, Publication

## Abstract

*Journal of Hematology & Oncology* is pleased to see the exponential growth in the number of publications from China, especially in hematology and oncology. This editorial calls for putting more weight on the quality of the future scientific output and invites rigorous dialogs among policy makers, granting agencies, academic leaders, physicians, and scientists, followed by concrete actions, to achieve such a goal.

## Editorial

The number of scientific publications from China has exploded in the past 10 years. Multiple factors have propelled this exponential growth. Among these factors include but are not limited to a large scientific research base, increases in government funding through Natural Science Foundation of China, acute upholding of the publish-or-perish mantra, monetary reward for publications from local institutions, institutional requirement for publications in quality journals for awarding doctoral degrees, and faculty promotions. Dr. An-mei Deng’s group from Shanghai performed a detailed and focused analysis of hematology research output from China in the past 10 years [1]. The study compared and contrasted the quantities and qualities of publications from China with those from the United States, Great Britain, Germany, Japan, and South Korea. It is clear that China has become a high output country of scientific publications in the international journals, yet the quality disproportionally lagged behind.

China has succeeded in quantitative promotion of scientific research [2]. The next step is to focus on improving quality. This will require time and patience. To achieve this goal, it is imperative to continue to reform the education system to place the greatest emphasis on originality and creativity of the scientific endeavor in both clinical and basic research. A conducive environment must be preserved to put more weight on the quality of research rather than publication number in measuring scientific output. Funding decisions shall also be made not based on the trending topics in the field but strategically on nurturing the growth of scientific leaders and allowing time for high quality researches to mature. Even though the return of many top scientists to China from the United States and other countries has clearly enhanced the quality of scientific publications, cultivation of homegrown scientific leaders is the only sustainable way for China to lead in the scientific world.
